# Genomic characterisation of Chinese myeloid malignancies and its clinical correlates: insights from targeted next-generation sequencing

**DOI:** 10.3389/fmed.2026.1773307

**Published:** 2026-04-30

**Authors:** Pengjun Liao, Ruohao Xu, Suxia Geng, Chengxin Deng, Xin Huang, Ping Wu, Minming Li, Lingji Zeng, Peilong Lai, Jianyu Weng, Xin Du

**Affiliations:** Guangdong Provincial People’s Hospital, Guangdong Academy of Medical Sciences, Southern Medical University, Guangzhou, China

**Keywords:** acute myeloid leukemia, chronic myelomonocytic leukemia, disease progression, mutation, myelodysplastic syndromes, prognosis, SNP

## Abstract

**Purposes:**

Gene mutations identified through next-generation sequencing (NGS) have been increasingly recognised for their clinical significance in myeloid malignancies. However, data concerning Chinese populations remain limited. This study aimed to characterise mutation profiles in Chinese patients with myeloid malignancies and to evaluate the associations of genetic alterations with clinical features, disease progression, and prognosis.

**Methods:**

Targeted sequencing using a 110-gene panel was performed on 184 samples derived from 174 patients diagnosed with acute myeloid leukaemia (AML, *n* = 108), myelodysplastic syndromes (MDS, *n* = 66), and chronic myelomonocytic leukaemia (CMML, *n* = 10). Prognostic relevance of clinical variables, gene mutations, and single nucleotide polymorphisms (SNPs) was assessed using univariate and multivariate Cox regression analyses.

**Results:**

In newly diagnosed AML cases, the most frequently mutated genes included *FLT3* (19%), *NPM1* (19%), *DNMT3A* (17%), and *NRAS* (17%). In MDS, mutations in *DNMT3A* (21%), *TP53* (21%), and *TET2* (19%) were most prevalent. Among CMML patients, *ASXL1* and *SRSF2* mutations were observed in 50%, and *RUNX1* in 40% of cases. A significantly higher incidence of *WT1* mutations was observed in relapsed AML compared with newly diagnosed cases. In patients with secondary AML transformed from CMML, *NPM1* and *FAT1* mutations occurred at significantly higher frequencies than in CMML alone. Specific SNPs in *FAT1*, *PDGFRA*, *SETBP1*, and *KMT2C* were associated with poorer prognosis in AML. In MDS, *TP53* mutations, *FAT1*/ *IL7R* SNPs, and elevated peripheral blood blast percentages were identified as adverse prognostic indicators.

**Conclusion:**

Targeted NGS revealed distinct mutational landscapes in Chinese patients with AML, MDS, and CMML. These findings demonstrated associations between genetic alterations and clinical features, disease progression, and prognosis in Chinese patients with myeloid malignancies. However, the prognostic associations should be interpreted cautiously, as none of the evaluated variables remained independent prognostic factors in multivariate models.

## 1 Introduction

Myeloid malignancies are heterogenous clonal diseases originated from myeloid hematopoietic progenitor cells, with acute myeloid leukemia (AML) as the most common form followed by myelodysplastic syndrome (MDS) and myeloproliferative neoplasm (MPN) (1). MDS/MPN is a rare myeloid malignancies with overlapping features from MPN and MDS, of which chronic myelomonocytic leukemia (CMML) is the most frequent occurrence, characteristic by the presence of sustained peripheral blood monocytosis with recurrent mutations (2). It is widely acknowledged that myeloid malignancies are highly heterogeneous in molecular characteristics, where gene mutations assume pivotal roles. With the advancement of technology, next-generation sequencing (NGS), especially the targeted sequencing, is being widely used for genomic characterization of clinical samples. Identification of molecular abnormalities through NGS can provide extensive information on diagnosis, prognosis and treatment in patients with myeloid malignancies (3). To date, the genetic landscapes of Western patients with myeloid malignancies have been comprehensively reported, but reports on the mutation pattern in Chinese patients are limited.

Among the myeloid malignancies, AML has the highest mortality rate, with a five-year survival rate of about 40% (1). Relapse is one of the major reasons for its low survival rate. Both MDS and CMML are chronic myeloid malignancies with higher survival rates compared with AML (4). However, unfortunately, about one-third MDS and CMML patients tend to transform into secondary AML (sAML) and the transformation is the main cause of lethal outcomes in these patients (5, 6). Relapse and transformation are considered as disease progression, which may be triggered by clonal evolution as cytogenetic and molecular alteration occurred in almost every transformed case (7, 8). Previous studies have revealed gene alterations that play a role in disease relapse and transformation in Western patients (9). For example, Ding et al. (10) indicated that mutations in *WAC*, *SMC3*, *DIS3*, *DDX41*, and *DAXX* were frequent in relapsed AML. Lee et al. (11) found that mutations of SF3B1, SRSF2, ASXL1, TET2, and DNMT3A conferred an increased risk for MDS evolution to leukemia. However, similar studies on the association of genetic mutations with disease relapse and transformation have rarely been conducted in Chinese populations and the marker genes predicting disease relapse and transformation of myeloid malignancies in Chinese patients are lacking.

Moreover, gene mutations are strongly linked to the prognosis. Many studies found that genes such as *TP53*, *RUNX1*, and *ASXL1* mutations were associated with poor outcomes in myeloid malignancies (12, 13). Several studies have suggested that selected single nucleotide polymorphisms may be associated with clinical outcome in myeloid malignancies. For example, IDH1 c.315C>T and TET2 rs2454206 have been reported to correlate with prognosis in AML. On this basis, we exploratorily evaluated the potential prognostic associations of both recurrent gene mutations and selected SNPs in patients with myeloid malignancies (14, 15). Therefore, we explored the prognostic effects of both mutant genes and SNP in patients with myeloid malignancies.

In our study, we first retrospectively analyzed the molecular landscapes of baseline Chinese patients with myeloid malignancies, and patterns of co-occurrence and mutual exclusivity of mutant genes and SNP. Subsequently, we explored the associations between gene mutations and clinical features and disease progression. Finally, we investigated the prognostic significance of mutations and SNP.

## 2 Methods

### 2.1 Clinical samples

A total of 174 patients from the Institute of Guangdong Provincial People’s Hospital between December 2019 and February 2023 were enrolled and sorted in this study, including 101 AML (2 transformed from the MDS cases during treatment), 63 MDS and 10 CMML. AML, MDS and CMML were diagnosed according to the criteria of the criterion of World Health Organization (WHO) (16). Clinic data including age, sex, red blood cells (RBC), white blood cells (WBC), hemoglobin (Hb), platelet (PLT), lactic dehydrogenase (LDH), bone marrow (BM) blast percentage, peripheral blood (PB) blast percentage, BM WT1 expression, and PB WT1 expression were collected from the medical records. Samples from patients with sufficient bone marrow (BM) at diagnosis, or during treatment, or at relapse were collected for genomic analysis. Informed consents were obtained from all patients and this study was carried out in compliance with the Declaration of Helsinki. This study was approved by the ethics committee of Institute of Guangdong Provincial People’s Hospital (伦理号).

### 2.2 Targeted sequencing

Genomic DNA (gDNA) was extracted from the BM using the gDNA Extraction Kit (TIANGEN, Beijing, China). In general, 200 ng gDNA was used to create the sequencing library targeting 110 genes (listed in Supplementary Table 1) (Rightongene, Shanghai, China) using MultipSeq Custom Panel. The library construction process was as follows: first, gDNA was amplificated by PCR using multiple primers, and the amplified products were combined and purified by AMPure XP magnetic beads (Beckman, California, USA); then, purified products were amplificated by PCR using adapter primers and purified by magnetic beads again; finally, the library concentration was recorded by Qubit 3.0 Flourometer (Thermo, Massachusetts, USA) and the length and purity of the library fragment were measured by Qsep100 automated nucleic acid protein analysis system (BIOptic, Jiangsu, China). Targeted NGS was performed on the Novaseq platform (Illumina, California, USA).

### 2.3 Cytogenetic-related methods

Conventional karyotyping and FISH results were retrieved from the clinical records. Karyotypes were categorized as normal, abnormal, or complex karyotype according to routine cytogenetic reports. FISH positivity was defined as the presence of at least one abnormal signal detected by the clinically applied probe set used in routine diagnostic evaluation. Because FISH detects cytogenetic abnormalities rather than sequence-level variants, FISH/karyotype findings were analyzed as clinicopathologic variables rather than as direct molecular equivalents of NGS-detected point mutations.

### 2.4 Bioinformatic analysis

Original sequencing image data file was converted into the sequencing data through base recognition and stored as FASTQ file. The quality of the original sequencing data was evaluated by FastQC (version 1.11.4) software. Trimmatic (version 3.6) software was used to remove joint information, low-quality bases, or undetected bases. After sorting and eliminating repetitive sequences, BAM files were obtained. The data of BAM files including library average length, comparison rate, coverage, capture rate, sequencing depth, homogeneity was used to evaluate the quality of sample library. Based on the BAM results, the SNP and InDel sites were detected by GATK and Mutet2 and annotated by ANNOVAR. SNPs were defined using the ExAC and 1,000 Genomes project with >0.1% population frequency. Matched germline samples were not available in this study. Therefore, somatic mutations could not be definitively distinguished from rare germline variants, particularly in the interpretation of SNP-related findings, although population databases and annotation-based filtering were applied.

In addition, mutations meeting the following criteria were filtered out: (1) SNP; (2) variant allele frequency (VAF) <5%; and (3) mutations predicted as harmless by SIFT and PolyPhen2. This filtering strategy was used to reduce potential false-positive low-level calls, but may also have excluded low-frequency subclonal variants. The final retained variants may be deleterious, likely deleterious or unknown significance, and were included in the mutation profile analyses.

### 2.5 Statistical analysis

Statistical analysis was performed using R package (version 4.1.2). Quantitative data was compared by *t*-test, or Mann–Whitney U test. Categorical variables were evaluated using the Fisher’s exact test followed by Bonferroni correction. Overall survival (OS) was defined as the time from the diagnosis to the last follow-up or death due to any cause. Progression-free survival (PFS) was defined as the time from the diagnosis to last follow-up or the disease progression (relapse or death). SNP-related survival analyses were performed as exploratory analyses to identify candidate associations for future validation. Because multiple exploratory comparisons were performed, particularly in the co-occurrence and SNP-related analyses, not all analyses were adjusted for multiple testing. Therefore, nominal *p* values from these exploratory analyses should be interpreted with caution. The co-occurrence and SNP-related analyses were intended to identify candidate associations for future validation rather than to support definitive inference.

Analyses involving WT1 expression and laboratory variables were performed based on available cases because some variables had missing data. All tests were two-tailed, *p* < 0.05 was considered to be significantly different. * represents *p* < 0.05 and ** represents *p* < 0.01. Graphs were made in Prism 8, v8.2.0 (GraphPad Software Inc.) and Adobe Illustrator 2021 (Adobe Inc.).

## 3 Results

### 3.1 Overview of cohort characteristics

A total of 184 samples were obtained from 174 patient with WHO defined myeloid malignancies, including 108 AML (75 at diagnosis, 25 during treatment, 8 at relapse), 66 MDS (43 at diagnosis, 21 during treatment, 2 at relapse) and 10 CMML (at diagnosis), were observed in the study. The clinical features of all patients were summarized in Tables 1–3. Of 108 AML samples, 14 (13.0%) cases were diagnosed with sAML (11 transformed from the MDS and 3 transformed from the CMML). The median ages of AML, MDS and CMML groups were 53, 66, and 65.5 years, respectively. According to the European LeukemiaNet (ELN) 2022 criteria, 22 (29.3%), 28 (37.3%), and 25 (33.3%) AML patients were classified in the favorable, intermediate, and adverse risk group. Based on the International Prognostic Scoring System (IPSS-R), 2 (4.7%), 11 (25.6%), 17 (39.5%), and 13 (30.2%) MDS patients were categorized into low, intermediate, high, and very high-risk group.

**Table 1:** Clinical characteristics of 108 cases with acute myeloid leukemia.

Variable	AML total (*n* = 108)	Newly diagnosed (*n* = 75)	During treatment (*n* = 25)	Relapsed (*n* = 8)
Female, *n* (%)	53 (46.5)	31 (41.3)	16 (64)	5 (62.5)
Age, median (range), y	54 (21–79)	52 (21–79)	61 (27–77)	55.5 (33–73)
Disease status, *n* (%)				
Primary	94 (87.0)	66 (88)	20 (80)	8 (100)
Secondary	14 (13.0)	9 (12)	5 (20)	0
Karyotyping, *n* (%)				
Normal karyotype	70 (69.3)	48 (69.6)	17 (70.8)	5 (62.5)
Abnormal karyotype	22 (21.8)	18 (26.1)	2 (8.3)	2 (25)
Complex karyotype	9 (8.9)	3 (4.3)	5 (20.8)	1 (12.5)
Missing date, *n*	7	6	1	0
FISH, *n* (%)				
Negative	73 (63.2)	47 (69.1)	18 (81.8)	8 (100)
Positive	25 (8.8)	21 (30.9)	4 (18.2)	0 (0)
Missing date, *n*	10	7	3	0
RBC, median (range), ×10^12^ /L	2.44 (1.01–799.0)	2.34 (1.01–608)	2.68 (1.72–799)	3.065 (2.1–4.31)
WBC, median (range), ×10^9^ /L	5.5 (0.50–432.29)	12.78 (0.79–432.29)	3.82 (0.5–141.2)	2.55 (1.38–2.255)
Hb, median (range), g/L	76 (36–149)	74 (36–149)	79 (49–138)	95.5 (67–134)
PLT, median (range), x10^9^/L	45.5 (4–1,510)	38 (5–1,510)	58 (4–515)	97.5 (50–310)
LDH, median (range), U/L	334 (110–2,142)	483.5 (115–2,142)	189 (110–799)	235 (174–1,211)
Missing date, *n*	2	2		
BM blast percentage, median (range), %	49 (1.5–94)	59 (7–97)	25.5 (1.5–91)	22.5 (5–75)
Missing date, *n*	5	4	1	0
PB blast percentage, median (range), %	25.5 (0–96)	35 (1–96)	3 (0–91)	3 (0–53)
Missing date, n	12	9		
BM WT1 expression, median (range), (10^4^ /ABL)	3388.83 (0.21–17954.29)	3770.45 (0.21–15459.13)	2402.35 (0.47–17954.29)	4923.655 (11.7–15783.6)
Missing date, *n*	11	9	2	0
PB WT1 expression, median (range), (10^4^ /ABL)	3,960 (0–40084.75)	5161.52 (0–28391.16)	870.22 (0–40084.75)	720.655 (60.85–7881.06)
Missing date, *n*	57	43	12	2
ELN risk classification				
Favorable		22 (29.3)		
Intermediate		28 (37.3)		
Adverse		25 (33.3)		

RBC, red blood cells, WBC, white blood cells; Hb, hemoglobin; PLT, platelet; LDH, lactic dehydrogenase; BM, Bone marrow; PB, Peripheral blood; ELN, European LeukemiaNet.

**Table 2:** Clinical characteristics of 66 cases with myelodysplastic syndrome.

Variable	MDS total (*n* = 66)	Newly diagnosed (*n* = 43)	During treatment (*n* = 21)	Relapsed (*n* = 2)
Female, *n* (%)	21 (31.8)	12 (27.9)	7 (33.3)	2 (100)
Age, median (range), y	64 (21–87)	62 (21–87)	65 (47–75)	51 (33–69)
Karyotyping, *n* (%)				
Normal karyotype	30 (52.6)	15 (41.7)	15 (78.9)	0
Abnormal karyotype	20 (35.1)	15 (41.7)	3 (15.8)	2 (100)
Complex karyotype	7 (12.3)	6 (16.7)	1 (5.3)	0
Missing date, *n*	9	7	2	0
FISH, *n* (%)				
Negative	28 (46.7)	14 (35)	13 (72.2)	1 (50)
Positive	32 (53.3)	26 (65)	5 (27.8)	1 (50)
Missing date, *n*	6	3	3	0
RBC, median (range), ×10^12^ /L	2.16 (0.92–354)	2.16 (0.92–354)	2.16 (1.6–3.86)	2.22 (1.48–2.96)
WBC, median (range), ×10^9^ /L	2.48 (0.27–20.99)	2.22 (0.59–20.99)	3.24 (0.27–9.02)	2.565 (2.48–2.65)
Missing date, *n*	1	1	0	0
Hb, median (range), g/L	66 (17–120)	65 (36–113)	68 (17–120)	78 (51–105)
Missing date, *n*	1	1	0	0
PLT, median (range), x10^9^/L	76 (2–581)	80 (8–581)	51 (2–439)	141.5 (115–168)
LDH, median (range), U/L	212 (2.18–735)	206 (2.18–735)	208 (113–533)	251.5 (221–282)
Missing date, *n*	2	2	0	0
BM blast percentage, median (range), %	5 (0.5–24)	6 (0.5–24)	2.5 (0.5–14)	9 (6–12)
Missing date, *n*	2	1	1	0
PB blast percentage, median (range), %	1 (0–18)	1 (0–18)	0 (0–8)	2 (2–2)
Missing date, *n*	6	2	4	0
BM WT1 expression, median (range), (10^4^ /ABL)	415.88 (0.49–8810.8)	648.905 (4.79–8810.8)	197.08 (0.49–2381.88)	1658.745 (915.17–2402.32)
Missing date, *n*	5	3	2	0
PB WT1 expression, median (range), (10^4^ /ABL)	110.06 (0.38–4977.97)	209.47 (0.38–4977.97)	30.93 (0.79–878.36)	2330.2 (2330.2–2330.2)
Missing date, *n*	27	19	7	1
IPSS-R risk group				
Very low		0 (0)		
Low		2 (4.7)		
Intermediate		11 (25.6)		
High		17 (39.5)		
Very high		13 (30.2)		

RBC, red blood cells, WBC, white blood cells; Hb, hemoglobin; PLT, platelet; LDH, lactic dehydrogenase; BM, Bone marrow; PB, Peripheral blood; IPSS, International Prognostic Scoring System.

**Table 3:** Clinical characteristics of 10 cases with chronic myelomonocytic leukemia.

Variable	CMML (*n* = 10)
Female, *n* (%)	5 (50)
Age, median (range), y	65.5 (22–74)
Karyotyping, *n* (%)	
Normal karyotype	5 (55.6)
Abnormal karyotype	3 (33.3)
Complex karyotype	1 (11.1)
Missing date, *n*	1
FISH, *n* (%)	
Negative	8 (80)
Positive	2 (20)
RBC, median (range), ×10^12^ /L	2.73 (1.65–3.7)
WBC, median (range), ×10^9^ /L	9.19 (4.21–24.93)
Hb, median (range), g/L	73 (61–104)
PLT, median (range), ×10^9^/L	44 (5–515)
LDH, median (range), U/L	206 (164–562)
BM blast percentage, median (range), %	6.5 (2.5–15.6)
PB blast percentage, median (range), %	2 (0–13)
BM WT1 expression, median (range), (10^4^ /ABL)	211.49 (8.93–15732.19)
PB WT1 expression, median (range), (10^4^ /ABL)	251.46 (23.38–965.35)
Missing date, *n*	3

### 3.2 Landscapes of genetic mutations in myeloid malignancies

**Figure 1:**
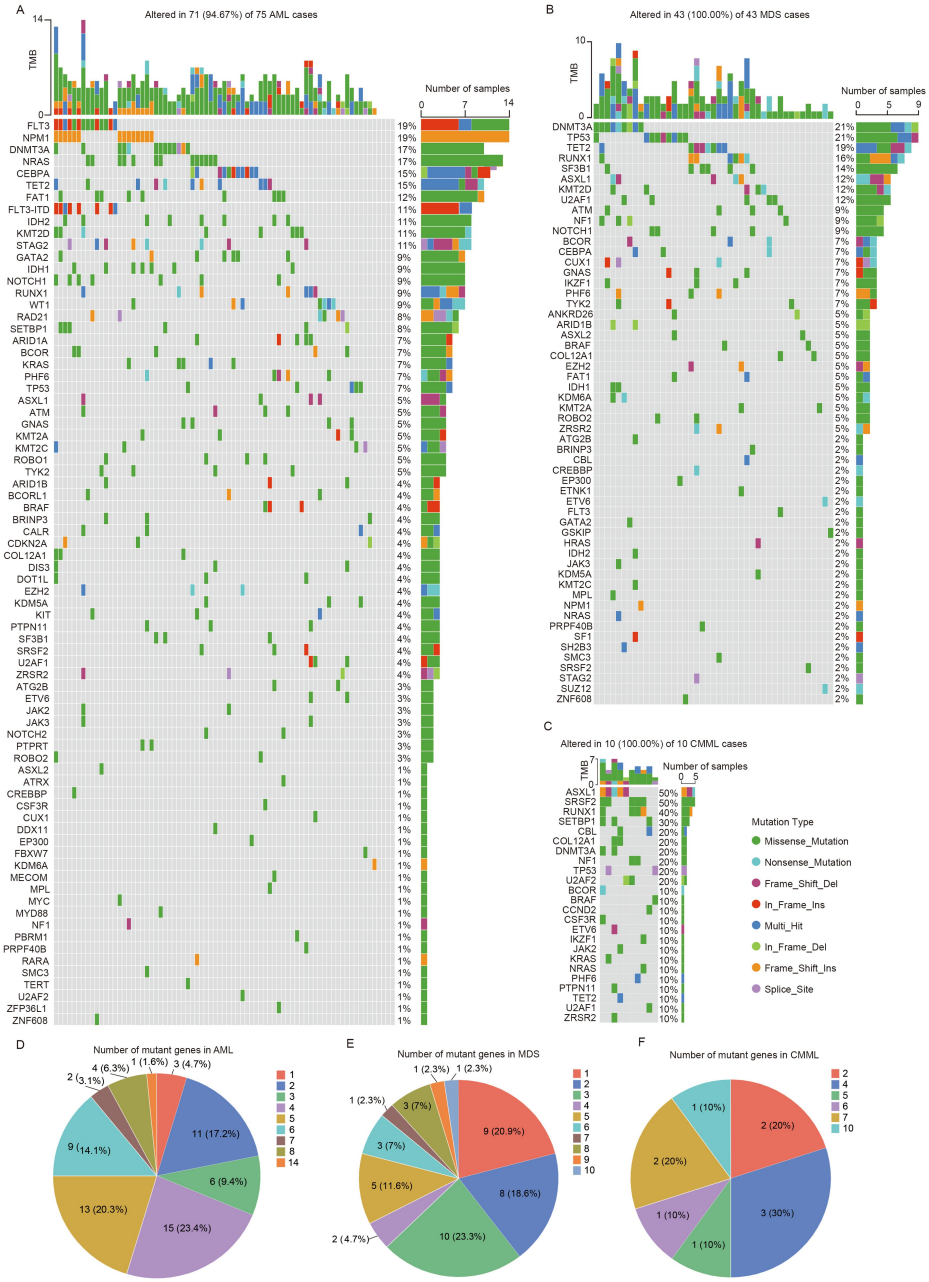
Gene mutation landscapes of newly diagnosed patients with myeloid malignancies. Genetic mutation patterns in AML **(A)**, MDS **(B)**, and CMML **(C)**. Distribution of the number of mutations per patient in AML **(D)**, MDS **(E)**, and CMML **(F)**.

The targeted panel NGS was performed in all the patients and the mutation landscapes of newly diagnosed patients were showed in Figure 1. It could be indicated that a total of 75 mutated genes and 331 mutation were detected in 94.67% (71/75) of the AML patients, among which the most frequently mutated gene was *FLT3* (19%, *FLT3-ITD*, 11%) and *NPM1* (19%), followed by *DNMT3A* (17%), *NRAS* (17%), *CEBPA* (15%), and *TET2* (15%) (Figure 1A). Meanwhile, a total of 56 mutated genes and 156 mutations were observe in 100% (43/43) of the MDS patients, and the most common molecular events in the MDS patients were *DNMT3A* (21%) and *TP53* (21%) mutations, followed by *TET2* (19%), *RUNX1* (16%), *SF3B1* (14%), *ASXL1* (12%), *KMT2D* (12%) and *U2AF1* (12%) mutations (Figure 1B). As for the CMML patients, 10 cases had a total of 24 mutated genes and 51 mutations, where the most common mutated genes were *ASXL1* (50%) *and SRSF2* (50%), followed by *RUNX1* (40%), *SETBP1* (30%), *CBL* (20%), *COL12A1* (20%), *DNMT3A* (20%), *NF1* (20%), *TP53* (20%), and *U2AF2* (20%) (Figure 1C). Among patients harboring at least one mutation, the median number of mutations per sample was 4 (range 1–14), 3 (range 1–10), and 4.5 (range 2–10) in AML, MDS, and CMML, respectively. In AML and MDS, most patients carried one to five mutations, whereas only a minority harbored more than seven mutations (Figures 1D–F). We included this metric as a descriptive indicator of the overall distribution of mutational burden across disease groups, rather than as a validated marker of clinical stratification or prognosis (Figures 1D–F).

**Figure 2:**
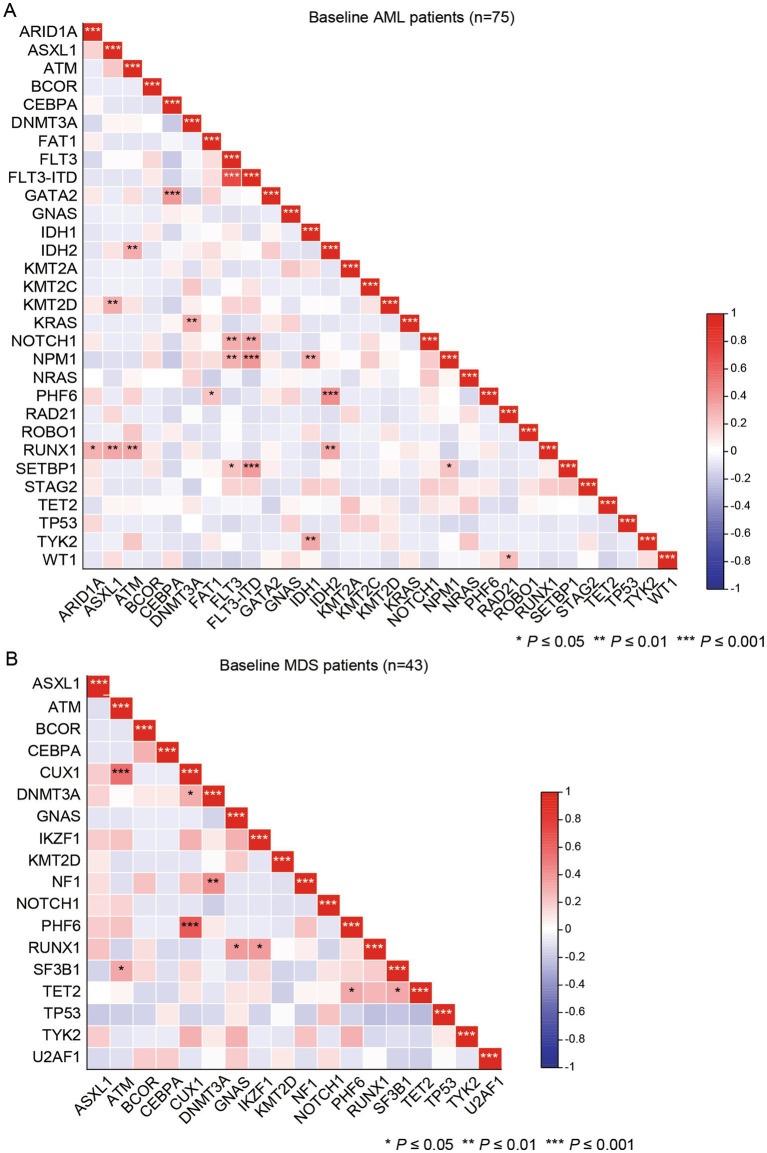
The patterns of co-occurrence and mutual exclusivity of mutant genes in AML **(A)** and MDS **(B)** patients.

Next, we explored the patterns of co-occurrence and mutual exclusivity of mutant genes (mutational frequency ≥5%). In AML patients, *FLT3-ITD* mutations were highly associated with *NPM1* (*p* < 0.001), *SETBP1* (*p* < 0.001), and *NOTCH1* (*p* < 0.01) mutations. *CEBPA* mutations were frequently co-mutated with *GATA2* (*p* < 0.001) mutations, and *RUNX1* mutations had strong associations with *IDH2* (*p* < 0.01), *ASXL1* (*p* < 0.01), *ATM* (*p* < 0.01) and *ARID1A* (*p* < 0.05, Figure 2A). Regarding MDS patients, we observed that *CUX1* mutations were strongly related to *ATM* (*p* < 0.001) and *PHF6* (*p* < 0.001) mutations, and *DNMT3A* mutations had closely associations with *NF1* (*p* < 0.001) mutations (Figure 2B). Moreover, we investigated the co-occurrence and mutual exclusivity of SNP with mutant genes in AML and MDS patients (Supplementary Tables 2, 3). For the co-occurrence and mutual exclusivity of mutations and SNPs in the same gene, we only found strong correlations between *EZH2* mutation and *EZH2* SNP rs2302427 (*p* < 0.05), and between *TET2* mutations and *TET2* SNP rs2454206 (*p* < 0.05) in AML and MDS patients, respectively. This indicated that there may be no link between mutations and SNPs in the majority of genes. Regarding the co-occurrence and mutual exclusivity of mutations and SNPs in the different gene, it’s worth noting that the *TP53* mutations in AML patients had strong association with *FAT1* SNP rs146085516 (*p* < 0.001), *FAT1* SNP rs1280097/rs2637777/rs75367100, *ATM* SNP rs659243, *DIS3* SNP rs4883918, *ATG2B* SNP rs3759601, *SETBP1* SNP rs3744825, and *KMT2C* SNP rs4024453 (*p* < 0.05). In MDS patients, *TP53* mutations were significantly correlated with *TET2* SNP rs12498609, *FAT1* SNP rs77834784/rs3796648/rs2304867, and *KMT2C* SNP rs199504848 (*p* < 0.05). These findings suggested that these SNPs were associated with TP53 mutations in this cohort. However, because multiple co-occurrence and SNP-related comparisons were examined, these findings may be subject to false-positive results and require further validation. On the contrary, mutated *TP53* was significantly exclusive with the *SMARCA2* SNP rs2296212 (*p* < 0.05) in MDS patients, indicating that the prognosis of patients with that SNP may not be affected by malignant mutations of *TP53*.

### 3.3 Correlations of gene mutations with clinical features

**Figure 3:**
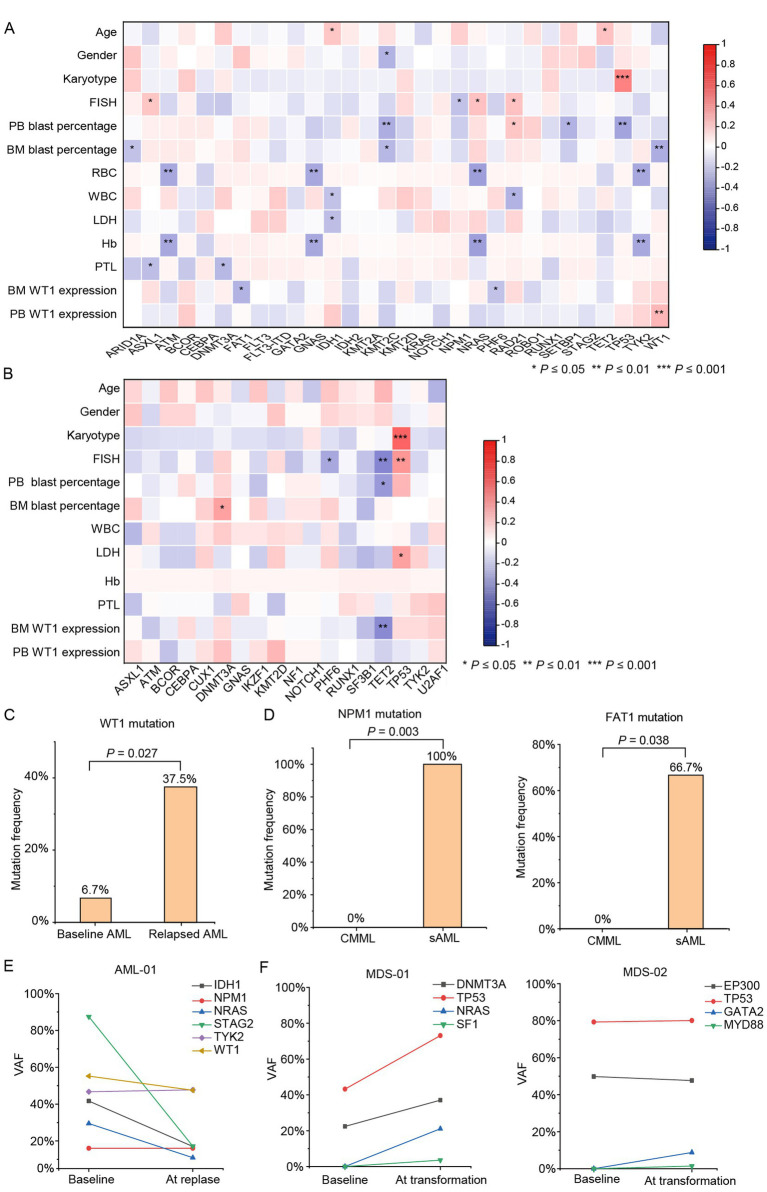
The correlations of gene mutation with clinical features and disease relapse and transformation. The significant relationship between gene mutations and clinical characteristics in AML **(A)** and MDS **(B)**. Genes with significantly different mutation frequencies in newly diagnosed AML/relapse AML **(C)** and primary AML/sAML (transformed from CMML) **(D)**. Longitudinal mutation profiles and observed VAF changes of AML-01 **(E)**, MDS-01 and MDS-02 **(F)** during disease progression.

Next, we explored the associations between gene mutations and clinical characteristics in different disease groups. In the AML cohort, *TET2* and *IDH1* mutations (*p* < 0.05) were more common in elderly patients. *NPM1* mutations (*p* < 0.05) occurred preferentially in FISH negative patients, whereas *ASXL1*, *NRAS*, and *RAD21* mutations (*p* < 0.05) were more common in FISH positive patients. *TP53* mutation (*p* < 0.001) was associated with complex karyotype. Patients with *IDH1* mutations (*p* < 0.05) tended to have normal LDH level. In addition, *ARID1A* (*p* < 0.05), *KMT2C* (*p* < 0.05), and *WT1* (*p* < 0.01) mutations were correlated with lower BM blast percentage, while *KMT2C* (*p* < 0.01), *TP53* (*p* < 0.01) and *SETBP1* (*p* < 0.05) mutations were association with lower PB blast percentage. Notably, *WT1* mutations (*p* < 0.01) were strongly associated with PB WT1 expression (Figure 3A).

In the MDS cohort, similar to the AML cohort, *TP53* mutation (*p* < 0.001) was also associated with complex karyotype. *TET2* (*p* < 0.01) and *PHF6* (*p* < 0.05) mutations were significantly implicated in FISH negative patients, whereas *TP53* (*p* < 0.01) mutation was common in FISH negative patients. Patients with *TP53* mutations (*p* < 0.05) were inclined to have abnormal PLT level. *DNMT3A* mutation (*p* < 0.05) and *TET2* mutation (*p* < 0.05) was correlated with higher BM blast percentage and lower PB blast percentage, respectively. *TET2* (*p* < 0.01) mutation was association with lower WT1 expression in BM. In addition, there was no statistically significant difference in MDS patients when investigating the relationship between gene mutations and gender, age, WBC, Hb, PTL, and WT1 expression in PB (Figure 3B).

### 3.4 Correlations of gene mutations with disease relapse and transformation

We further studied the correlation between gene mutation (number of mutated genes, number of mutated sites and VAF of mutated genes) and relapse and transformation in newly diagnosed AML/relapsed AML, primary AML (pAML)/sAML (transformed from MDS and CMML), MDS/sAML (transformed from MDS) and CMML/sAML (transformed from CMML). After inter-group comparisons, WT1 mutation appeared to be more frequent in relapsed AML (37.5% vs. 6.7%, *p* = 0.027, *n* = 8 vs. *n* = 75) than in newly diagnosed AML (Figure 3C). In addition, NPM1 and FAT1 mutations were observed more frequently in sAML transformed from CMML than in CMML at diagnosis (Figure 3D). However, because these comparisons were based on very small subgroups, particularly the CMML-derived sAML subgroup, these findings should be interpreted cautiously as preliminary observations. No significant correlation was found between gene mutations and disease progression in pAML/sAML or MDS/sAML transformed from MDS. No significant correlation was found between the gene mutations and disease progression in pAML (*n* = 66) /sAML (*n* = 9) and MDS (*n* = 43) /sAML (transformed from MDS, *n* = 6).

In our cohort, eight patients were sampled twice, of which three patients were sampled at initial diagnosis and disease relapse or transformation, respectively. The first patient (AML-01) was diagnosed as *de novo* AML at initial diagnosis, and was completely relieved after treatment, but relapsed 2 years later. Mutations of *NPM1*, *NRAS*, *IDH1*, *WT1*, *STAG2*, and *TYK2* were detected in AML-01 both at diagnosis and at relapse. Notably, NPM1 and TYK2 showed higher observed VAF at relapse than at diagnosis, whereas the remaining genes showed lower observed VAF (Figure 3E). However, because VAF may be influenced by tumor burden and related factors, these differences should not be interpreted as definitive evidence of clonal dominance or clonal architecture (Figure 3E). In addition, the second patient (MDS-01) and the third patient (MDS-02) was diagnosed as MDS firstly, but transformed to AML 15 months and 9 months after treatment, respectively. Of note, apart from the mutated genes detected at diagnosis, several new mutant genes were detected in MDS-01 (*NRAS* and *SF1* mutations) and MDS-02 (*MYD88* and *GATA2* mutations) at the transformation of MDS to AML (Figure 3F). These longitudinal observations suggested dynamic changes in mutation profiles during disease progression in these individual cases. However, given the very limited number of paired samples and the lack of adjustment for tumor burden or copy-number status, these findings should be considered preliminary and should not be overinterpreted in terms of clonal architecture.

### 3.5 Survival analysis

**Figure 4:**
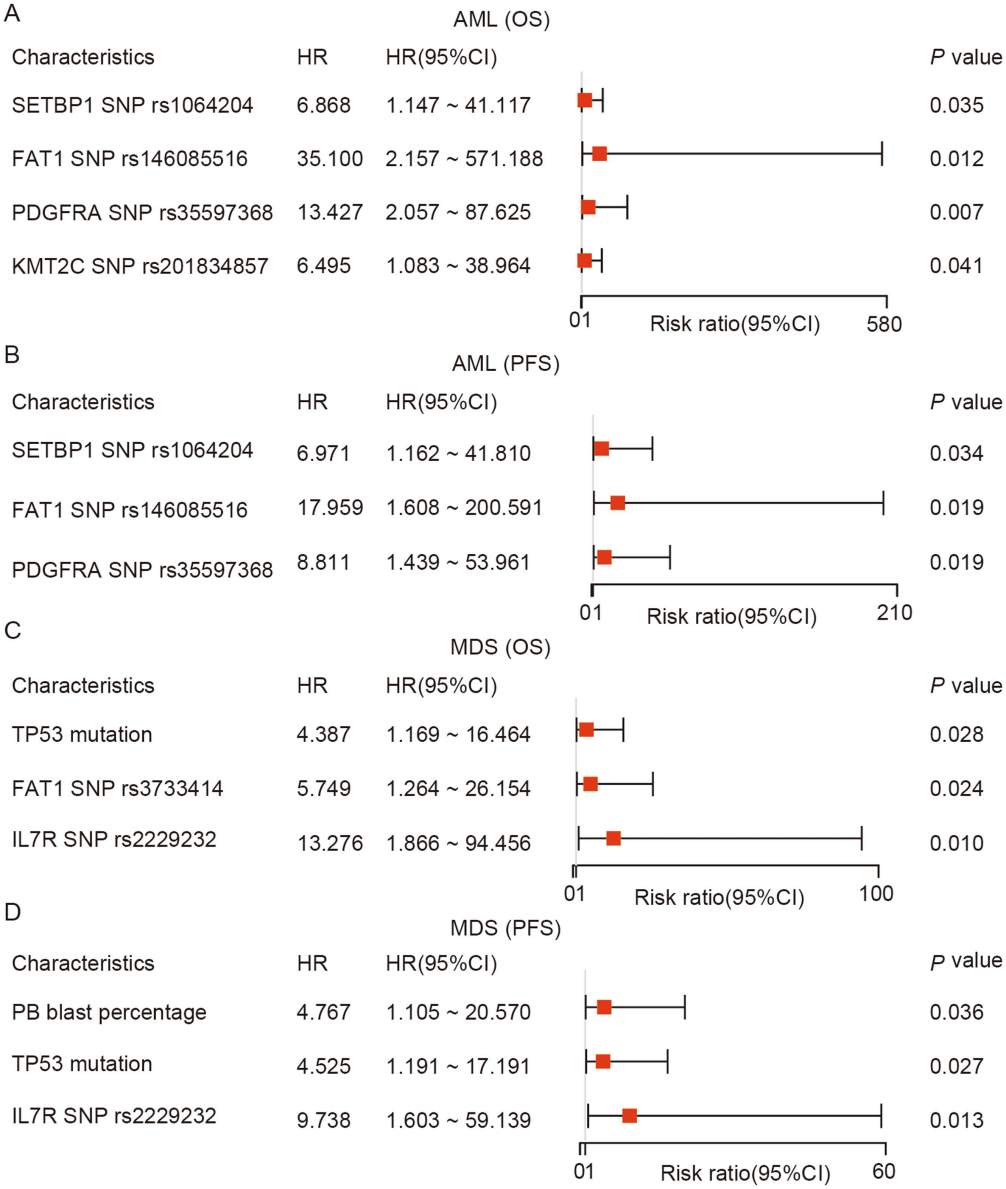
Exploratory prognostic analyses in baseline AML and MDS patients. Forest plots showing univariate associations of selected clinical and genetic variables with OS and PFS in AML **(A,B)** and MDS **(C,D)**.

Finally, univariate and multivariate analyses of the prognostic effects for variables including gene mutations (mutational frequency ≥5%), number of mutated genes, number of mutated sites, VAF of mutated genes, SNPs and clinical information (age, sex, RBC, WBC, Hb, PLT, LDH, BM blast percentage, PB blast percentage, BM WT1 expression, and PB WT1 expression) from baseline AML and MDS patients was conducted (Supplementary Tables 4, 5). The prognostic analyses involving SNPs were exploratory, and associations involving low-frequency variants were interpreted with particular caution. The median follow-up for AML patients and MDS patients was 11.3 and 15.7 months, respectively. The results showed that AML patients with *PDGFRA* SNP rs35597368 (*p* = 0.007), *FAT1* SNP rs146085516 (*p* = 0.012), *SETBP1* SNP rs1064204 (*p* = 0.035), and *KMT2C* SNP rs201834857 had significantly shorter OS (Figure 4A). Meanwhile, *PDGFRA* SNP rs35597368 (*p* = 0.019), *FAT1* SNP rs146085516 (*p* = 0.019), and *SETBP1* SNP rs1064204 (*p* = 0.034) also indicated the significantly inferior PFS in AML patients (Figure 4B). For the MDS patients, *TP53* (*p* = 0.028), *FAT1* SNP rs3733414 (*p* = 0.024), and *IL7R* SNP rs2229232 (*p =* 0.01) mutations were significantly related to shorter OS (Figure 4C). Additionally, *TP53* mutation (*p* = 0.027), *IL7R* SNP rs2229232 (*p* = 0.01), and high PB blast percentage (*p* = 0.045) were significantly associated with shorter PFS (Figure 4D). The multivariate analyses showed that none of these variables remained independent prognostic factors. Therefore, the above prognostic associations should be interpreted cautiously and regarded as exploratory findings.

## 4 Discussion

In this study, we provide a single-center descriptive genomic profiling dataset of AML, MDS, and CMML in a Chinese cohort. The mutational landscape observed in our cohort was largely consistent with previous reports in both Western and Chinese populations. In addition, we exploratorily assessed the associations of genetic alterations with disease relapse, transformation, and clinical outcome. These clinicogenomic and prognostic findings should be interpreted as supplementary and exploratory rather than as definitive novel discoveries.

Over the past few years, the molecular heterogeneity of myeloid malignancies has been extensively characterized in large Western cohorts (17–20), and several studies in Chinese populations have also reported recurrent mutation patterns broadly similar to those observed in Western patients (21–23). Consistent with these reports, the most frequently mutated genes in our cohort included NPM1, DNMT3A, FLT3, and NRAS in AML; SF3B1, TET2, ASXL1, and SRSF2 in MDS; and TET2, SRSF2, and ASXL1 in CMML. These findings suggest that our study mainly provides complementary population-specific data from a Chinese single-center cohort, rather than a fundamentally distinct mutational landscape. In addition, we presented the distribution of the number of mutations per patient as a descriptive measure of overall genomic complexity across disease groups. However, the clinical or prognostic significance of this metric was not specifically established in the present study and therefore should not be overinterpreted.

In our study, we analyzed the association between clinical features and mutation information in patients with AML or MDS. We confirmed that the frequency of *TET2* mutation was significantly higher in AML patients aged ≥ 60 years than younger patients, which was consistent with previous literature (24). *TP53* mutation is known to be associated with complex karyotype in both AML and MDS (25, 26), which was also demonstrated by our study. Of note, Heesch et al. (27) found that *WT1* mutation cases were characterized by higher *WT1* expression in adult acute T-lymphoblastic leukemia. The same phenomenon was also observed in our AML cohort, suggesting that in certain diseases, *WT1* mutation and expression may be closely associated. In addition, several gene mutation frequencies were significantly higher in AML and MDS patients with normal blood levels or FISH-negative. This finding revealed that mutations in several genes may precede abnormal clinical manifestations among these patients. In other words, it may be that certain gene mutations lead to abnormalities in clinical features, which suggested that there was a period of latency that preceded obvious abnormal clinical features during which early detection and monitoring could be considered (28, 29). Because FISH and conventional karyotyping detect cytogenetic abnormalities rather than sequence-level variants, these findings should be interpreted as associations between different layers of genomic aberration rather than direct molecular concordance.

Most AML patients die from progressive disease after relapse, which is associated with dynamic clonal evolution (10). With the widespread use of NGS, we have known that the mutational profile at relapse is frequently discrepant from that at diagnosis because mutations are frequently gained or lost during the course of the disease. Furthermore, the observed VAFs of gene mutations may change during the course of the disease. However, without adjustment for tumor burden or copy-number status, such changes should be interpreted cautiously and cannot be taken as definitive evidence of clonal architecture or biological dominance (30). In our study, WT1 mutation appeared more frequent in relapsed AML than in newly diagnosed AML, and WT1 mutation was also observed at relapse in one paired AML case. These findings were broadly consistent with previous reports, but should be interpreted cautiously because the relapsed AML subgroup was very small (31).

Approximately 30% of MDS and CMML patients will experience an AML evolution. Transformation of MDS and CMML to AML is one of the largest contributors of mortality in those patients (32–34). By using NGS, many retrospective case series have suggested that genetic alterations were contributors to leukemic transformation and several genetic mutations frequently implicated in the leukemic transformation were identified. For example, Xu et al. (35) demonstrated that the accumulation of mutations in genes related to epigenetic modifications (RNA splicing, DNA methylation, chromatin modification, signal transduction, transcriptional regulation) represented an important factor in the MDS transformation to AML, in which *TET2*, *IDH1*, and *IDH2* mutations were driving events and were acquired with the transformation of MDS to AML. Geissler et al. (36) reported that mutations in genes relevant to the RAS signaling pathway (such as *NRAS*, *KRAS*, *NF-1*, *PTPN11*, and *CBL*) were later acquired and were driver mutations for CMML-derived sAML. Makishima et al. (37) proved that *SETBP1* mutation seemed to be gain-of-function, and was associated with an increased risk of sAML development. Taken together, these studies revealed that AML cells routinely acquired additional mutations at transformation. In our cohort, NPM1 and FAT1 mutations were observed in CMML-derived sAML. However, because this observation was based on an extremely small number of transformed cases, it should be regarded as preliminary and should not be interpreted as evidence of a definitive driving role in transformation (38). Due to the number of samples, we did not find significant difference of number of mutated genes, number of mutated sites and VAF of mutated genes between MDS and sAML patients. However, we found that mutations of *NRAS* and *SF1* appeared in MDS-01 and *MYD88* and *GATA2* in MDS-02 at relapse, which were not detected at diagnosis. This result indicated that those mutations acquired later during leukemia transformation and might cause the final transformation.

It is increasingly clear that different gene mutations have different impact on prognosis in myeloid malignancies. Numerous previous studies have demonstrated that *DNMT3A*, *RUNX1*, *ASXL1*, *SRSF2*, *TP53* and *SF3B1* mutations predict poor outcomes in AML patients (24, 39). Moreover, *NPM1*, *TP53*, *KRAS*, *RUNX1*, *NF1*, *GATA2* and *ASXL1* mutations are associated with poor prognosis, whereas *SF3B1* mutation has a positive impact on survival in MDS patients (40, 41). However, our study did not draw those conclusions regarding prognosis, which may be due to the limited number of cases and complexity of factors affecting prognosis. Notably, not only did we study the prognostic effects of mutations in patients, we also examined the prognostic significance of SNP. In this cohort, FAT1 SNP rs146085516 was associated with inferior survival in AML and was also found to co-occur with TP53 mutation. Importantly, although several variables were associated with OS or PFS in univariate analyses, none remained independent prognostic factors in multivariate models. Given the limited sample size and the wide confidence intervals of the survival estimates, this finding should be considered exploratory and hypothesis-generating rather than clinically applicable. The relatively small sample size reduced the precision and stability of the prognostic estimates. Moreover, several SNP-related hazard ratios were accompanied by wide confidence intervals, indicating limited statistical stability and warranting cautious interpretation. Because a large number of statistical tests were performed, particularly in the co-occurrence and SNP-related analyses, and not all exploratory analyses were adjusted for multiple comparisons, some nominally significant findings may reflect false-positive associations. In addition, some mutation- or SNP-related associations may have been influenced by very low-frequency or single-event variants, which can inflate apparent prognostic effects in a small cohort and further limit interpretability. Larger independent cohorts are required to validate these findings.

In addition, matched germline samples were not available, which limited our ability to definitively distinguish somatic mutations from rare germline variants, particularly in the SNP analyses. Although population frequency databases and annotation-based filtering were applied, residual misclassification cannot be completely excluded.

In conclusion, our study found that mutant hotspot genes of myeloid malignancies in Chinese patients were similar with those in Western patients. Notably, frequencies of *WT1*, and *NPM1* and *FAT1* mutations were found to be significantly higher in AML patients who relapsed and transformed from CMML, respectively. In addition, our study identified several SNPs that were associated with adverse outcomes in exploratory univariate analyses of AML and MDS, although none remained independent prognostic factors in multivariate models. These findings provide descriptive and exploratory, population-specific data on the molecular landscape of myeloid malignancies in Chinese patients and offer complementary evidence to the existing literature, but require further validation before any clinical application. At last, the use of a VAF cutoff of 5% may have excluded clinically relevant low-frequency subclonal mutations, particularly in the analyses of disease progression and relapse, and may therefore have led to underestimation of subclonal complexity. The findings related to relapse and transformation were derived from very small subgroups and should therefore be considered preliminary observations requiring validation in larger cohorts.

## Data Availability

The datasets generated and/or analyzed in the current study include human sequencing data. Due to ethical approval restrictions and participant privacy considerations, the raw sequencing data cannot be made publicly available in an open repository. De-identified data relevant to the findings of this study may be made available to qualified researchers upon reasonable request, subject to approval by the institutional ethics committee and in accordance with institutional policies and applicable regulations. Requests to access the datasets should be directed to the corresponding authors at xind_gdph2020@163.com.

## References

[1] Shallis RM Wang R Davidoff A Ma X Zeidan AM. Epidemiology of acute myeloid leukemia: recent progress and enduring challenges. Blood Rev. (2019) 36:70–87. doi: 10.1016/j.blre.2019.04.005, 31101526

[2] Patnaik MM Lasho T. Myelodysplastic syndrome/myeloproliferative neoplasm overlap syndromes: a focused review. Hematology Am Soc Hematol Educ Program. (2020) 2020:460–4. doi: 10.1182/hematology.2020000163, 33275673 PMC7727594

[3] Fukuhara S Oshikawa-Kumade Y Kogure Y Shingaki S Kariyazono H Kikukawa Y . Feasibility and clinical utility of comprehensive genomic profiling of hematological malignancies. Cancer Sci. (2022) 113:2763–77. doi: 10.1111/cas.15427, 35579198 PMC9357666

[4] Bewersdorf JP Zeidan AM. Risk-adapted, individualized treatment strategies of myelodysplastic syndromes (MDS) and chronic Myelomonocytic leukemia (CMML). Cancers (Basel). (2021) 13:1610. doi: 10.3390/cancers13071610, 33807279 PMC8036734

[5] Belotserkovskaya E Demidov O. Mouse models of CMML. Int J Mol Sci. (2021) 22:11510. doi: 10.3390/ijms222111510, 34768940 PMC8584008

[6] Reinig E Yang F Traer E Arora R Brown S Rattray R . Targeted next-generation sequencing in myelodysplastic syndrome and chronic Myelomonocytic leukemia aids diagnosis in challenging cases and identifies frequent spliceosome mutations in transformed acute myeloid leukemia. Am J Clin Pathol. (2016) 145:497–506. doi: 10.1093/ajcp/aqw016, 27124934

[7] Kishtagari A Levine RL Viny AD. Driver mutations in acute myeloid leukemia. Curr Opin Hematol. (2020) 27:49–57. doi: 10.1097/MOH.0000000000000567, 31972687 PMC11104430

[8] Bănescu C Tripon F Muntean C. The genetic landscape of myelodysplastic neoplasm progression to acute myeloid leukemia. Int J Mol Sci. (2023) 24:5734. doi: 10.3390/ijms24065734, 36982819 PMC10058431

[9] Menssen AJ Walter MJ. Genetics of progression from MDS to secondary leukemia. Blood. (2020) 136:50–60. doi: 10.1182/blood.2019000942, 32430504 PMC7332895

[10] Ding L Ley TJ Larson DE Miller CA Koboldt DC Welch JS . Clonal evolution in relapsed acute myeloid leukaemia revealed by whole-genome sequencing. Nature. (2012) 481:506–10. doi: 10.1038/nature10738, 22237025 PMC3267864

[11] Lee P Yim R Fung SH Miu KK Wang Z Wu KC . Single-nucleotide variations, insertions/deletions and copy number variations in myelodysplastic syndrome during disease progression revealed by a single-cell DNA sequencing platform. Int J Mol Sci. (2022) 23:4647. doi: 10.3390/ijms23094647 35563039 PMC9100947

[12] Döhner H Wei AH Appelbaum FR Craddock C DiNardo CD Dombret H . Diagnosis and management of AML in adults: 2022 recommendations from an international expert panel on behalf of the ELN. Blood. (2022) 140:1345–77. doi: 10.1182/blood.2022016867 35797463

[13] Garcia-Manero G. Myelodysplastic syndromes: 2023 update on diagnosis, risk-stratification, and management. Am J Hematol. (2023) 98:1307–25. doi: 10.1002/ajh.26984, 37288607 PMC12002404

[14] Corley EM Mustafa Ali MK Alharthy H Kline KAF Sewell D Law JY . Impact of IDH1 c.315C>T SNP on outcomes in acute myeloid leukemia: a propensity score-adjusted cohort study. Front Oncol. (2022) 12:804961. doi: 10.3389/fonc.2022.804961, 35372066 PMC8972959

[15] Wang X Chen X Yang Z Dou H Lu L Bi J . Correlation of TET2 SNP rs2454206 with improved survival in children with acute myeloid leukemia featuring intermediate-risk cytogenetics. Genes Chromosomes Cancer. (2018) 57:379–86. doi: 10.1002/gcc.22540, 29664232

[16] Khoury JD Solary E Abla O Akkari Y Alaggio R Apperley JF . The 5th edition of the World Health Organization classification of Haematolymphoid Tumours: myeloid and histiocytic/dendritic neoplasms. Leukemia. (2022) 36:1703–19. doi: 10.1038/s41375-022-01613-1, 35732831 PMC9252913

[17] Papaemmanuil E Gerstung M Bullinger L Gaidzik VI Paschka P Roberts ND . Genomic classification and prognosis in acute myeloid leukemia. N Engl J Med. (2016) 374:2209–21. doi: 10.1056/NEJMoa1516192, 27276561 PMC4979995

[18] Ogawa S. Genetics of MDS. Blood. (2019) 133:1049–59. doi: 10.1182/blood-2018-10-844621, 30670442 PMC6587668

[19] Ley TJ Miller C Ding L Raphael BJ Mungall AJ Robertson A . Genomic and epigenomic landscapes of adult de novo acute myeloid leukemia. N Engl J Med. (2013) 368:2059–74. doi: 10.1056/NEJMoa1301689, 23634996 PMC3767041

[20] Patnaik MM Tefferi A. Chronic myelomonocytic leukemia: 2022 update on diagnosis, risk stratification, and management. Am J Hematol. (2022) 97:352–72. doi: 10.1002/ajh.26455, 34985762

[21] Wang RQ Chen CJ Jing Y Qin JY Li Y Chen GF . Characteristics and prognostic significance of genetic mutations in acute myeloid leukemia based on a targeted next-generation sequencing technique. Cancer Med. (2020) 9:8457–67. doi: 10.1002/cam4.3467, 32970934 PMC7666719

[22] Hou HA Tsai CH Lin CC Chou WC Kuo YY Liu CY . Incorporation of mutations in five genes in the revised international prognostic scoring system can improve risk stratification in the patients with myelodysplastic syndrome. Blood Cancer J. (2018) 8:39. doi: 10.1038/s41408-018-0074-7, 29618722 PMC5884776

[23] Nie Y Shao L Zhang H He CK Li H Zou J . Mutational landscape of chronic myelomonocytic leukemia in Chinese patients. Exp Hematol Oncol. (2022) 11:32. doi: 10.1186/s40164-022-00284-z, 35610628 PMC9128105

[24] Metzeler KH Herold T Rothenberg-Thurley M Amler S Sauerland MC Görlich D . Spectrum and prognostic relevance of driver gene mutations in acute myeloid leukemia. Blood. (2016) 128:686–98. doi: 10.1182/blood-2016-01-693879, 27288520

[25] Weinberg OK Siddon A Madanat YF Gagan J Arber DA Dal Cin P . TP53 mutation defines a unique subgroup within complex karyotype de novo and therapy-related MDS/AML. Blood Adv. (2022) 6:2847–53. doi: 10.1182/bloodadvances.2021006239, 35073573 PMC9092405

[26] Geng S Xu R Huang X Li M Deng C Lai P . Dynamics of PD-1 expression are associated with treatment efficacy and prognosis in patients with intermediate/high-risk myelodysplastic syndromes under hypomethylating treatment. Front Immunol. (2022) 13:950134. doi: 10.3389/fimmu.2022.950134, 36003379 PMC9393298

[27] Heesch S Goekbuget N Stroux A Tanchez JO Schlee C Burmeister T . Prognostic implications of mutations and expression of the Wilms tumor 1 (WT1) gene in adult acute T-lymphoblastic leukemia. Haematologica. (2010) 95:942–9. doi: 10.3324/haematol.2009.016386, 20435628 PMC2878792

[28] Desai P Mencia-Trinchant N Savenkov O Simon MS Cheang G Lee S . Somatic mutations precede acute myeloid leukemia years before diagnosis. Nat Med. (2018) 24:1015–23. doi: 10.1038/s41591-018-0081-z, 29988143 PMC6849383

[29] Xu R Wu M Wang Y Li C Zeng L Wang Y . Mesenchymal stem cells reversibly de-differentiate myofibroblasts to fibroblast-like cells by inhibiting the TGF-β-SMAD2/3 pathway. Mol Med. (2023) 29:59. doi: 10.1186/s10020-023-00630-9, 37098464 PMC10131436

[30] Thol F Ganser A. Treatment of relapsed acute myeloid leukemia. Curr Treat Options in Oncol. (2020) 21:66. doi: 10.1007/s11864-020-00765-5, 32601974 PMC7324428

[31] El Hussein S DiNardo CD Takahashi K Khoury JD Fang H Furudate K . Acquired WT1 mutations contribute to relapse of NPM1-mutated acute myeloid leukemia following allogeneic hematopoietic stem cell transplant. Bone Marrow Transplant. (2022) 57:34992253:370–6. doi: 10.1038/s41409-021-01538-w 34992253

[32] Tsai SC Shih LY Liang ST Huang YJ Kuo MC Huang CF . Biological activities of RUNX1 mutants predict secondary acute leukemia transformation from chronic Myelomonocytic leukemia and myelodysplastic syndromes. Clin Cancer Res. (2015) 21:3541–51. doi: 10.1158/1078-0432.CCR-14-2203, 25840971

[33] Inoue D Kitaura J Matsui H Hou HA Chou WC Nagamachi A . SETBP1 mutations drive leukemic transformation in ASXL1-mutated MDS. Leukemia. (2015) 29:847–57. doi: 10.1038/leu.2014.301, 25306901 PMC4501574

[34] Xu R Huang X Li C Deng C Li M Wu P . Bone marrow mesenchymal stromal cells in chronic myelomonocytic leukaemia: overactivated WNT/β-catenin signalling by parallel RNA sequencing and dysfunctional phenotypes. Br J Haematol. (2021) 193:928–40. doi: 10.1111/bjh.17425, 33959953

[35] Xu F Wu LY Guo J He Q Zhang Z Li X. Somatic mutations of activating signalling, transcription factor, and tumour suppressor are a precondition for leukaemia transformation in myelodysplastic syndromes. J Cell Mol Med. (2022) 26:5901–16. doi: 10.1111/jcmm.17613, 36380727 PMC9716205

[36] Geissler K Jäger E Barna A Gurbisz M Graf T Graf E . Correlation of RAS-pathway mutations and spontaneous myeloid Colony growth with progression and transformation in chronic Myelomonocytic leukemia-a retrospective analysis in 337 patients. Int J Mol Sci. (2020) 21:3025. doi: 10.3390/ijms21083025, 32344757 PMC7215883

[37] Makishima H Yoshida K Nguyen N Przychodzen B Sanada M Okuno Y . Somatic SETBP1 mutations in myeloid malignancies. Nat Genet. (2013) 45:942–6. doi: 10.1038/ng.2696, 23832012 PMC3729750

[38] Wu P Weng J Li M Lu Z Deng C Sun Q . Co-occurrence of RUNX1 and ASXL1 mutations underlie poor response and outcome for MDS patients treated with HMAs. Am J Transl Res. (2019) 11:3651–8. 31312376 PMC6614648

[39] Han X Li W He N Feng P Pang Y Ji C . Gene mutation patterns of Chinese acute myeloid leukemia patients by targeted next-generation sequencing and bioinformatic analysis. Clin Chim Acta. (2018) 479:25–37. doi: 10.1016/j.cca.2018.01.006, 29309772

[40] Chiereghin C Travaglino E Zampini M Saba E Saitta C Riva E . The genetics of myelodysplastic syndromes: clinical relevance. Genes (Basel). (2021) 12:1144. doi: 10.3390/genes12081144, 34440317 PMC8392119

[41] Nazha A. The MDS genomics-prognosis symbiosis. Hematology Am Soc Hematol Educ Program. (2018) 2018:270–6. doi: 10.1182/asheducation-2018.1.270, 30504321 PMC6246025

